# Waist Circumference as Compared with Body-Mass Index in Predicting Mortality from Specific Causes

**DOI:** 10.1371/journal.pone.0018582

**Published:** 2011-04-26

**Authors:** Michael F. Leitzmann, Steven C. Moore, Annemarie Koster, Tamara B. Harris, Yikyung Park, Albert Hollenbeck, Arthur Schatzkin

**Affiliations:** 1 Nutritional Epidemiology Branch, Division of Cancer Epidemiology and Genetics, National Cancer Institute, National Institutes of Health, U.S. Department of Health and Human Services, Bethesda, Maryland, United States of America; 2 Department of Epidemiology and Preventive Medicine, Regensburg University Medical Center, Regensburg, Germany; 3 Laboratory of Epidemiology, Demography, and Biometry, Intramural Research Program, National Institute on Aging, National Institutes of Health, U.S. Department of Health and Human Services, Bethesda, Maryland, United States of America; 4 Department of Internal Medicine, School for Public Health and Primary Care (CAPHRI), Maastricht University, Maastricht, The Netherlands; 5 AARP, Washington, D.C., United States of America; University of Pittsburgh Medical Center, United States of America

## Abstract

**Background:**

Whether waist circumference provides clinically meaningful information not delivered by body-mass index regarding prediction of cause-specific death is uncertain.

**Methods:**

We prospectively examined waist circumference (WC) and body-mass index (BMI) in relation to cause-specific death in 225,712 U.S. women and men. Cox regression was used to estimate relative risks and 95% confidence intervals (CI). Statistical analyses were conducted using SAS version 9.1.

**Results:**

During follow-up from 1996 through 2005, we documented 20,977 deaths. Increased WC consistently predicted risk of death due to any cause as well as major causes of death, including deaths from cancer, cardiovascular disease, and non-cancer/non-cardiovascular diseases, independent of BMI, age, sex, race/ethnicity, smoking status, and alcohol intake. When WC and BMI were mutually adjusted in a model, WC was related to 1.37 fold increased risk of death from any cancer and 1.82 fold increase risk of death from cardiovascular disease, comparing the highest versus lowest WC categories. Importantly, WC, but not BMI showed statistically significant positive associations with deaths from lung cancer and chronic respiratory disease. Participants in the highest versus lowest WC category had a relative risk of death from lung cancer of 1.77 (95% CI, 1.41 to 2.23) and of death from chronic respiratory disease of 2.77 (95% CI, 1.95 to 3.95). In contrast, subjects in the highest versus lowest BMI category had a relative risk of death from lung cancer of 0.94 (95% CI, 0.75 to 1.17) and of death from chronic respiratory disease of 1.18 (95% CI, 0.89 to 1.56).

**Conclusions:**

Increased abdominal fat measured by WC was related to a higher risk of deaths from major specific causes, including deaths from lung cancer and chronic respiratory disease, independent of BMI.

## Introduction

Body-mass index (body weight in kilograms divided by the square of height in meters, BMI) is currently the most frequently used and widely accepted method to classify medical risk according to weight status. BMI is a useful measure of adiposity in young and middle-aged adults [1]. However, an important limitation of the BMI is its inability to distinguish between fat mass and fat-free mass, which show opposing relations with health risk [2]. In addition, the validity of BMI as an indicator of fatness in elderly persons is limited [3] because fat-free mass decreases with aging, even without a change in overall weight [4].

In contrast, waist circumference (WC) represents a measure of adiposity that takes into account the accumulation of abdominal fat. WC is simple to measure and interpret and it is highly correlated with visceral fat as assessed by computed tomography [5]. WC has increased significantly in United States (U.S.) women and men in recent decades, a secular trend that mirrors the development of generalized adiposity in this country. The potentially greater relevance of WC than BMI for predicting adverse health conditions in the elderly is underscored by the observation that fat mass accumulates intra-abdominally with age [6]. Older age groups have experienced a greater increase in abdominal obesity than younger age groups [7].

In a large, contemporary cohort of U.S. women and men, we examined the relations of general and abdominal adiposity to death from specific causes. Our study differs from most previous investigations in quantifying the dose-response associations according to both WC and BMI.

## Materials and Methods

### Study population

The NIH-AARP Diet and Health Study began in 1995–1996 when 566,407 AARP members aged 50 to 71 years and residing in one of six U.S. states (CA, FL, LA, NJ, NC, and PA) or two metropolitan areas (Atlanta, GA, and Detroit, MI) satisfactorily completed a baseline questionnaire on their medical history, current body weight, height, smoking habits, and diet [8]. In 1996–1997, a second questionnaire requesting additional information on abdominal circumference was sent to baseline questionnaire respondents within six months and was returned by 59.5 percent of participants. The Special Studies Institutional Review Board of the U.S. National Cancer Institute accepted return of the questionnaires as an indication of voluntary participation, in lieu of signed consent.

### Population for analysis

To incorporate the WC data collected on the second questionnaire, our analysis included the 234,182 potentially eligible participants who provided complete information regarding their WC on the second questionnaire. We excluded individuals with a BMI less than 18.5 kg/m^2^ (n = 2,720) because very low weight measured at older age is likely to reflect leanness as a consequence of chronic illness. We also excluded participants with a BMI greater than 60 (n = 65). We excluded subjects with missing information on smoking (n = 2,548). We also excluded persons with extreme values (beyond three inter-quartile ranges of the 75^th^ and 25^th^ percentiles) for WC (n = 3,137). The remaining analytical cohort comprised of 225,712 subjects.

### Cohort follow-up

Cohort members were followed-up from November 8, 1996 through December 31, 2005 by annual linkage of the cohort to the National Change of Address database maintained by the U.S. Postal Service and through processing of undeliverable mail, other address change update services, and directly from cohort members' notifications. For matching purposes, we have virtually complete data on first and last name, address history, gender, and date of birth. Social Security number is available for 85 percent of our cohort.

### Mortality ascertainment

Vital status is ascertained by annual linkage to the Social Security Administration Death Master File in the U.S. [9] Verification of vital status and cause of death information is provided by follow-up searches of the National Death Index (NDI) Plus. We estimate that our mortality ascertainment is greater than 93 percent complete [9], [10]. Cohort maintenance also involves periodic linkage to the eleven state cancer registries serving our cohort [eight baseline states plus three most common states of re-location (TX, AZ, and NV) during follow-up] [11].

### Assessment of anthropometric variables

WC was assessed in the second questionnaire using a pictured instruction. Participants were requested to measure their waist with a tape measure one inch above the navel while standing and to report values to the nearest quarter inch. Participants for whom a tape measure was not available were asked to leave a blank response. Information on body weight and height was requested in the baseline questionnaire.

For the analyses of WC, participants were divided into the following categories: normal (women: less than 80 centimeters; men: less than 94 centimeters), action level 1 (women: 80 to 87 centimeters; men: 94 to 101 centimeters), and action level 2 (women: 88 centimeters or higher; men: 102 centimeters or higher). Those categories incorporate the WHO cut-points of 88 centimeters or higher for women and 102 centimeters or higher for men that define a large WC [12] and they correspond to the action levels proposed by Lean et al. [13] We also created a more extreme group of WC of 96 centimeters or higher for women and 118 centimeters or higher for men to investigate a broader range of WC in relation to death. The group of subjects with normal WC (less than 80 centimeters for women; less than 94 centimeters for men) served as the reference group.

Participants were divided into four BMI categories that correspond to the definitions of normal weight (18.5 to 24.9kg/m^2^), overweight (25.0 to 29.9 kg/m^2^), obesity class 1 (30.0 to 34.9 kg/m^2^), and obesity classes 2 or 3 (35 kg/m^2^ or greater) proposed by the World Health Organization (WHO) [14]. The group of subjects with a BMI of 18.5 to 24.9 kg/m^2^ served as the reference group.

Our categorizations of WC and BMI resulted in similar fractions of the at-risk population in the lowest WC and BMI categories and in similar fractions of the at-risk population in the highest WC and BMI categories.

In large cohorts similar to ours, comparisons of self-reported and measured WCs showed correlation coefficients of 0.89 in women and 0.95 in men [15]. The correlations between self-reported as compared with technician-measured BMI values have been found to be in the area of 0.94 for women and 0.92 for men [16].

### Statistical analysis

Cox proportional hazards regression [17] with person-time as the time scale was used to estimate the relative risks and corresponding 95 percent confidence intervals of death. Follow-up time was calculated from the return date of the second questionnaire until death from any cause or the end of study in December 31, 2005, whichever occurred first. Tests of the proportional hazards assumptions for exposures and covariates included in our models indicated no departures. Tests of linear trend were conducted by modeling the median values of WC or BMI categories as a single continuous variable, the coefficient for which was evaluated using a Wald test.

The multivariate models were adjusted for age, gender, race/ethnicity, smoking status, and alcohol intake. We did not adjust for physical activity because physical activity is an important determinant of body weight. We considered education level as a potential confounder but did not retain that variable in the final model because it failed to substantially alter beta coefficients in our primary exposures. The analyses of WC were additionally adjusted for BMI, thereby producing a variable reflecting mainly abdominal fat depots. We tested for and found no effect modification by gender and therefore show results for women and men combined. All reported P values are based on two-sided hypothesis tests. Statistical analyses were conducted using SAS version 9.1 (SAS Institute, Cary, NC).

## Results

During 1,961,011 person-years of follow-up, we documented 20,977 deaths. The mean (SD) ages at study entry and end of follow-up were 62.9 (5.3) and 71.7 (5.3) years, respectively. For men, the minimum and maximum WC sizes were 69.2 and 136.5 cm, respectively. For women, the minimum and maximum WC sizes were 50.8 and 138.4 cm, respectively. WC was positively correlated with BMI (r = 0.72). Participants with large WC or those with high BMI tended to have lower education levels, they were less likely to currently smoke, and they consumed less alcohol than their lean counterparts (data not shown).

Risk of death from any cause increased monotonically with higher values of WC (**Table 1**). Using participants with normal WC as the reference group, women with WC of 96 centimeters or higher and men with WC of 118 centimeters or higher showed a nearly 70 percent increased risk of death from any cause (multivariate relative risk = 1.68; 95 percent confidence interval, 1.55 to 1.81; P for trend<0.0001). The relation of BMI to death from any cause was of similar magnitude as that with WC. As compared with normal BMI, the multivariate relative risk of death from any cause for obesity classes 2 or 3 was 1.68 (95 percent confidence interval, 1.57 to 1.79, P for trend<0.0001).

**Table 1:** Relative risk of death from any cause according to waist circumference and body mass index.

	Death from any cause				
Waist circumference (cm)	<80 (women); <94 (men)	80–87 (women); 94–101 (men)	88–95 (women); 102–117 (men)	≥96 (women); ≥118 (men)	P for trend
Person-years	814,082	528,937	544,588	73,404	
Number of deaths	7,611	5,529	6,631	1,206	
Number of participants	93,212	60,898	62,987	8,616	
Age-adjusted relative risk	1.0	1.06 (1.03–1.10)	1.28 (1.24–1.32)	1.92 (1.81–2.05)	<0.0001
Multivariate relative risk	1.0	1.11 (1.07–1.16)	1.29 (1.24–1.35)	1.68 (1.55–1.81)	<0.0001
Body mass index (kg/m^2^)	18.5–24.9	25.0–29.9	30.0–34.9	≥35.0	
Person-years	778,409	836,885	269,391	76,326	
Number of deaths	7,792	8,786	3,330	1,069	
Number of participants	89,360	96,296	31,169	8,888	
Age-adjusted relative risk	1.0	0.97 (0.94–0.99)	1.22 (1.17–1.27)	1.65 (1.54–1.75)	<0.0001
Multivariate relative risk	1.0	0.99 (0.96–1.02)	1.24 (1.19–1.29)	1.68 (1.57–1.79)	<0.0001

The multivariate models used person-time as the underlying time metric and included the following covariates: age at entry (continuous), sex (male, female), race/ethnicity (white, black, Hispanic, Asian), smoking status (never, former, current), and alcohol intake (0, <1, <3, ≥3 drinks per day). The analysis of waist circumference was additionally adjusted for body-mass index (18.5–24.9, 25.0–29.9, 30.0–34.9, ≥35.0 kg/m^2^).

WC was significantly positively associated with death from cancer, most notably death from lung cancer (**Table 2**). The multivariate relative risk of lung cancer death comparing extreme categories of WC was 1.77 (95 percent confidence interval, 1.41 to 2.23, P for trend<0.0001). In contrast, the risk estimates relating BMI to death from lung cancer were below unity and the BMI category representing overweight showed a statistically significant inverse association (multivariate relative risk as compared with normal weight = 0.92; 95 percent confidence interval, 0.85 to 0.99). The P value for the test for trend was statistically non-significant at 0.16.

**Table 2:** Relative risk of death from cancer according to waist circumference and body mass index.

	Death from any cancer				
Waist circumference (cm)	<80 (women); <94 (men)	80–87 (women); 94–101 (men)	88–95 (women); 102–117 (men)	≥96 (women); ≥118 (men)	P for trend
Person-years	814,082	528,937	544,579	73,404	
Number of deaths	3,339	2,377	2,783	409	
Age-adjusted relative risk	1.0	1.05 (0.99–1.11)	1.22 (1.16–1.28)	1.45 (1.30–1.60)	<0.0001
Multivariate relative risk	1.0	1.08 (1.02–1.14)	1.22 (1.14–1.30)	1.37 (1.21–1.56)	<0.0001
Body mass index (kg/m^2^)	18.5–24.9	25.0–29.9	30.0–34.9	≥35.0	
Person-years	778,400	836,885	269,391	76,326	
Number of deaths	3,405	3,780	1,377	346	
Age-adjusted relative risk	1.0	0.98 (0.93–1.03)	1.17 (1.10–1.25)	1.18 (1.06–1.32)	<0.0001
Multivariate relative risk	1.0	1.01 (0.97–1.06)	1.22 (1.14–1.29)	1.25 (1.12–1.40)	<0.0001

	Death from lung cancer				
Waist circumference (cm)	<80 (women); <94 (men)	80–87 (women); 94–101 (men)	88–95 (women); 102–117 (men)	≥96 (women); ≥118 (men)	
Number of deaths	1,140	773	885	127	
Multivariate relative risk	1.0	1.12 (1.02–1.24)	1.37 (1.23–1.53)	1.77 (1.41–2.23)	<0.0001
Body mass index (kg/m^2^)	18.5–24.9	25.0–29.9	30.0–34.9	≥35.0	
Number of deaths	1,260	1,212	367	86	
Multivariate relative risk	1.0	0.92 (0.85–0.99)	0.94 (0.83–1.05)	0.94 (0.75–1.17)	<0.16

	Death from obesity-related cancers				
Waist circumference (cm)	<80 (women); <94 (men)	80–87 (women); 94–101 (men)	88–95 (women); 102–117 (men)	≥96 (women); ≥118 (men)	
Number of deaths	837	599	800	117	
Multivariate relative risk	1.0	0.99 (0.88–1.11)	1.11 (0.98–1.26)	1.10 (0.86–1.40)	0.25
Body mass index (kg/m^2^)	18.5–24.9	25.0–29.9	30.0–34.9	≥35.0	
Number of deaths	837	973	433	110	
Multivariate relative risk	1.0	1.15 (1.05–1.27)	1.63 (1.45–1.83)	1.52 (1.24–1.86)	<0.0001

	Death from all other cancers				
Waist circumference (cm)	<80 (women); <94 (men)	80–87 (women); 94–101 (men)	88–95 (women); 102–117 (men)	≥96 (women); ≥118 (men)	
Number of deaths	1,362	1,005	1,098	165	
Multivariate relative risk	1.0	1.10 (1.00–1.20)	1.16 (1.05–1.29)	1.33 (1.08–1.63)	<0.0001
Body mass index (kg/m^2^)	18.5–24.9	25.0–29.9	30.0–34.9	≥35.0	
Number of deaths	1,308	1,595	577	150	
Multivariate relative risk	1.0	1.02 (0.95–1.09)	1.23 (1.10–1.35)	1.36 (1.15–1.61)	0.0008

The multivariate models used person-time as the underlying time metric and included the following covariates: age at entry (continuous), sex (male, female), race/ethnicity (white, black, Hispanic, Asian), smoking status (never, former, current), and alcohol intake (0, <1, <3, ≥3 drinks per day). The analyses of waist circumference were additionally adjusted for body-mass index (18.5–24.9, 25.0–29.9, 30.0–34.9, ≥35.0 kg/m^2^). Obesity-related cancers include colon cancer, breast cancer, esophageal cancer, uterine cancer, ovarian cancer, kidney cancer, and pancreatic cancer.

Both WC and BMI were significantly positively related to death from cardiovascular disease (**Table 3**). The positive relations of high versus low categories of WC to death from any cardiovascular disease, death from coronary heart disease, and death from other cardiovascular disease (including death from stroke) were 23 percent, 31 percent, and 9 percent weaker, respectively, than those with high versus low categories of BMI.

**Table 3:** Relative risk of death from cardiovascular disease according to waist circumference and body mass index.

	Death from any cardiovascular disease				
Waist circumference (cm)	<80 (women); <94 (men)	80–87 (women); 94–101 (men)	88–95 (women); 102–117 (men)	≥96 (women); ≥118 (men)	P for trend
Person-years	814,082	528,937	544,579	73,404	
Number of deaths	2,241	1,818	2,149	460	
Age-adjusted relative risk	1.0	1.18 (1.11–1.26)	1.42 (1.34–1.51)	2.60 (2.35–2.88)	<0.0001
Multivariate relative risk	1.0	1.17 (1.09–1.25)	1.28 (1.18–1.38)	1.82 (1.59–2.08)	<0.0001
Body mass index (kg/m^2^)	18.5–24.9	25.0–29.9	30.0–34.9	≥35.0	
Person-years	778,400	836,885	269,391	76,326	
Number of deaths	2,160	2,922	1,173	413	
Age-adjusted relative risk	1.0	1.11 (1.05–1.18)	1.52 (1.42–1.63)	2.37 (2.13–2.63)	<0.0001
Multivariate relative risk	1.0	1.14 (1.08–1.21)	1.53 (1.43–1.65)	2.37 (2.13–2.64)	<0.0001

	Death from coronary heart disease				
Waist circumference (cm)	<80 (women); <94 (men)	80–87 (women); 94–101 (men)	88–95 (women); 102–117 (men)	≥96 (women); ≥118 (men)	
Number of deaths	1,405	1,105	1,293	270	
Multivariate relative risk	1.0	1.10 (1.01–1.20)	1.18 (1.07–1.30)	1.64 (1.38–1.95)	<0.0001
Body mass index (kg/m^2^)	18.5–24.9	25.0–29.9	30.0–34.9	≥35.0	
Number of deaths	1,263	1,833	734	243	
Multivariate relative risk	1.0	1.18 (1.10–1.27)	1.59 (1.45–1.74)	2.38 (2.07–2.73)	<0.0001

	Death from other cardiovascular disease				
Waist circumference (cm)	<80 (women); <94 (men)	80–87 (women); 94–101 (men)	88–95 (women); 102–117 (men)	≥96 (women); ≥118 (men)	
Number of deaths	836	713	856	190	
Multivariate relative risk	1.0	1.28 (1.15–1.42)	1.45 (1.28–1.64)	2.15 (1.74–2.65)	<0.0001
Body mass index (kg/m^2^)	18.5–24.9	25.0–29.9	30.0–34.9	≥35.0	
Number of deaths	897	1,089	439	170	
Multivariate relative risk	1.0	1.08 (0.99–1.18)	1.44 (1.29–1.62)	2.35 (1.99–2.78)	<0.0001

The multivariate models used person-time as the underlying time metric and included the following covariates: age at entry (continuous), sex (male, female), race/ethnicity (white, black, Hispanic, Asian), smoking status (never, former, current), and alcohol intake (0, <1, <3, ≥3 drinks per day). The analyses of waist circumference were additionally adjusted for body-mass index (18.5–24.9, 25.0–29.9, 30.0–34.9, ≥35.0 kg/m^2^).

WC showed a monotonically positive association with death from non-cancer/non-cardiovascular disease, particularly death from chronic respiratory disease (**Table 4**). The multivariate relative risk of death from chronic respiratory disease comparing extreme categories of WC was 2.77 (95 percent confidence interval, 1.95 to 3.95; P for trend<0.0001). By comparison, BMI showed a J-shaped relation with death from chronic respiratory disease, with strong inverse associations for overweight and class 1 obesity and a weak, statistically non-significant positive relation for the combination of classes 2 and 3 obesity (multivariate relative risk compared with normal weight = 1.18; 95 percent confidence interval, 0.89 to 1.56; P for inverse trend<0.0001). The only non-cancer/non-cardiovascular death endpoint that showed no statistically significant positive relation with WC was death from injuries (multivariate relative risk = 1.14; 95 percent confidence interval, 0.70 to 1.87; P for trend = 0.94) (**Table 5**).

**Table 4:** Relative risk of death from any non-cancer/non-cardiovascular diseases and from selected non-cancer/non-cardiovascular diseases according to waist circumference and body mass index.

	Death from any non-cancer/non-cardiovascular disease				
Waist circumference (cm)	<80 (women); <94 (men)	80–87 (women); 94–101 (men)	88–95 (women); 102–117 (men)	≥96 (women); ≥118 (men)	P for trend
Person-years	814,082	528,937	544,579	73,404	
Number of deaths	2,031	1,334	1,699	337	
Age-adjusted relative risk	1.0	0.96 (0.89–1.03)	1.22 (1.14–1.30)	2.01 (1.79–2.25)	<0.0001
Multivariate relative risk	1.0	1.10 (1.02–1.19)	1.46 (1.34–1.59)	2.03 (1.74–2.37)	<0.0001
Body mass index (kg/m^2^)	18.5–24.9	25.0–29.9	30.0–34.9	≥35.0	
Person-years	778,400	836,885	269,391	76,326	
Number of deaths	2,227	2,084	780	310	
Age-adjusted relative risk	1.0	0.81 (0.76–0.86)	1.01 (0.93–1.09)	1.69 (1.49–1.89)	<0.0001
Multivariate relative risk	1.0	0.82 (0.77–0.87)	1.01 (0.93–1.09)	1.67 (1.48–1.88)	<0.0001

	Death from chronic respiratory disease				
Waist circumference (cm)	<80 (women); <94 (men)	80–87 (women); 94–101 (men)	88–95 (women); 102–117 (men)	≥96 (women); ≥118 (men)	
Number of deaths	490	254	317	66	
Multivariate relative risk	1.0	1.07 (0.91–1.25)	1.71 (1.43–2.05)	2.77 (1.95–3.95)	<0.0001
Body mass index (kg/m^2^)	18.5–24.9	25.0–29.9	30.0–34.9	≥35.0	
Number of deaths	635	326	111	55	
Multivariate relative risk	1.0	0.49 (0.43–0.57)	0.56 (0.46–0.69)	1.18 (0.89–1.56)	<0.0001

	Death from acute respiratory/infectious disease				
Waist circumference (cm)	<80 (women); <94 (men)	80–87 (women); 94–101 (men)	88–95 (women); 102–117 (men)	≥96 (women); ≥118 (men)	
Number of deaths	227	164	184	36	
Multivariate relative risk	1.0	1.18 (0.95–1.46)	1.37 (1.07–1.76)	2.20 (1.39–3.49)	0.0006
Body mass index (kg/m^2^)	18.5–24.9	25.0–29.9	30.0–34.9	≥35.0	
Number of deaths	247	247	91	26	
Multivariate relative risk	1.0	0.88 (0.73–1.05)	1.04 (0.82–1.33)	1.21 (0.81–1.82)	0.56

The multivariate models used person-time as the underlying time metric and included the following covariates: age at entry (continuous), sex (male, female), race/ethnicity (white, black, Hispanic, Asian), smoking status (never, former, current), and alcohol intake (0, <1, <3, ≥3 drinks per day). The analyses of waist circumference were additionally adjusted for body-mass index (18.5–24.9, 25.0–29.9, 30.0–34.9, ≥35.0 kg/m^2^).

**Table 5:** Relative risk of death from selected non-cancer/non-cardiovascular diseases according to waist circumference and body mass index.

	Death from diabetes and kidney disease				
Waist circumference (cm)	<80 (women); <94 (men)	80–87 (women); 94–101 (men)	88–95 (women); 102–117 (men)	≥96 (women); ≥118 (men)	
Number of deaths	191	142	270	75	
Multivariate relative risk	1.0	1.03 (0.82–1.31)	1.64 (1.29–2.10)	2.42 (1.67–3.52)	<0.0001
Body mass index (kg/m^2^)	18.5–24.9	25.0–29.9	30.0–34.9	≥35.0	
Number of deaths	174	273	154	77	
Multivariate relative risk	1.0	1.26 (1.04–1.53)	2.29(1.84–2.85)	4.78 (3.64–6.27)	<0.0001

	Death from injuries				
Waist circumference (cm)	<80 (women); <94 (men)	80–87 (women); 94–101 (men)	88–95 (women); 102–117 (men)	≥96 (women); ≥118 (men)	
Number of deaths	260	168	166	30	
Multivariate relative risk	1.0	1.00 (0.81–1.23)	0.95 (0.74–1.22)	1.14 (0.70–1.87)	0.94
Body mass index (kg/m^2^)	18.5–24.9	25.0–29.9	30.0–34.9	≥35.0	
Number of deaths	246	258	89	31	
Multivariate relative risk	1.0	0.89 (0.74–1.06)	1.02 (0.79–1.30)	1.51 (1.03–2.19)	0.24

	Death from other non-cancer/non-cardiovascular diseases				
Waist circumference (cm)	<80 (women); <94 (men)	80–87 (women); 94–101 (men)	88–95 (women); 102–117 (men)	≥96 (women); ≥118 (men)	
Number of deaths	863	606	762	130	
Multivariate relative risk	1.0	1.13 (1.01–1.26)	1.48 (1.31–1.68)	1.91 (1.50–2.43)	<0.0001
Body mass index (kg/m^2^)	18.5–24.9	25.0–29.9	30.0–34.9	≥35.0	
Number of deaths	925	980	335	121	
Multivariate relative risk	1.0	0.92 (0.84–1.01)	1.04 (0.91–1.18)	1.56 (1.29–1.89)	0.006

The multivariate models used person-time as the underlying time metric and included the following covariates: age at entry (continuous), sex (male, female), race/ethnicity (white, black, Hispanic, Asian), smoking status (never, former, current), and alcohol intake (0, <1, <3, ≥3 drinks per day). The analyses of waist circumference were additionally adjusted for body-mass index (18.5–24.9, 25.0–29.9, 30.0–34.9, ≥35.0 kg/m^2^).

We examined the combined effects of WC and BMI on risk of death (**Table 6**). For this analysis, we collapsed the top two BMI categories. In the group of participants with BMI values between 25.0 and 29.9 kg/m^2^, high versus low WC predicted an increased risk of death from any cause and death from specific causes. Participants with BMI levels of 18.5 to 24.9 kg/m^2^ also showed positive relations of WC to risk of death from any cause and death from specific causes, but risk estimates failed to reach statistical significance for the highest WC category and were statistically significant only for the second to highest WC category. BMI levels of 30.0 kg/m^2^ or higher attenuated the positive relation of WC to death.

**Table 6:** Multivariate relative risk of death from any cause and death from specific causes according to joint categories of waist circumference and body mass index.

Variable	Waist circumference (cm)			
	<80 (women); <94 (men)	80–87 (women); 94–101 (men)	88–95 (women); 102–117 (men)	≥96 (women); ≥118 (men)
**Death from any cause**				
Person-years	575,301	160,084	42,325	690
Body mass index 18.5–24.9	1.0	1.11 (1.05–1.17)	1.37 (1.26–1.50)	1.65 (0.89–3.07)
Person-years	229,312	320,304	280,558	6,712
Body mass index 25.0–29.9	1.0	1.11 (1.05–1.17)	1.26 (1.19–1.34)	2.01 (1.67–2.42)
Person-years	9,468	48,550	221,696	66,001
Body mass index ≥30.0	1.0	0.89 (0.72–1.10)	1.09 (0.89–1.34)	1.49 (1.21–1.83)
**Death from cancer**				
Body mass index 18.5–24.9	1.0	1.10 (1.02–1.20)	1.22 (1.06–1.40)	1.84 (0.76–4.42)
Body mass index 25.0–29.9	1.0	1.07 (0.98–1.16)	1.19 (1.09–1.30)	1.45 (1.06–1.98)
Body mass index ≥30.0	1.0	0.82 (0.59–1.13)	1.01 (0.75–1.37)	1.11 (0.81–1.52)
**Death from cardiovascular disease**				
Body mass index 18.5–24.9	1.0	1.19 (1.08–1.32)	1.40 (1.19–1.66)	1.92 (0.62–5.96)
Body mass index 25.0–29.9	1.0	1.14 (1.04–1.25)	1.22 (1.11–1.35)	2.38 (1.74–3.25)
Body mass index ≥30.0	1.0	0.84 (0.59–1.19)	1.00 (0.72–1.38)	1.55 (1.11–2.16)
**Death from non-cancer/non-cardiovascular diseases**				
Body mass index 18.5–24.9	1.0	1.04 (0.94–1.15)	1.59 (1.37–1.85)	1.15 (0.29–4.60)
Body mass index 25.0–29.9	1.0	1.15 (1.02–1.29)	1.46 (1.30–1.65)	2.74 (1.95–3.87)
Body mass index ≥30.0	1.0	1.18 (0.71–1.97)	1.52 (0.94–2.47)	2.37 (1.45–3.87)
**Death from lung cancer**				
Body mass index 18.5–24.9	1.0	1.09 (0.96–1.25)	1.32 (1.07–1.63)	3.06 (0.98–9.50)
Body mass index 25.0–29.9	1.0	1.19 (1.02–1.39)	1.38 (1.18–1.62)	1.84 (1.13–2.98)
Body mass index ≥30.0	1.0	0.83 (0.42–1.64)	1.15 (0.61–2.17)	1.40 (0.73–2.68)
**Death from respiratory mortality**				
Body mass index 18.5–24.9	1.0	0.96 (0.79–1.17)	1.60 (1.22–2.08)	-
Body mass index 25.0–29.9	1.0	1.58 (1.12–2.21)	2.38 (1.71–3.31)	3.20 (1.35–7.57)
Body mass index ≥30.0	1.0	0.64 (0.18–2.32)	1.19 (0.38–3.77)	2.36 (0.74–7.56)

The multivariate models used person-time as the underlying time metric and included the following covariates: age at entry (continuous), sex (male, female), race/ethnicity (white, black, Hispanic, Asian), smoking status (never, former, current), and alcohol intake (0, <1, <3, ≥3 drinks per day). Participants with a waist circumference of <80 cm (women) or <94 cm (men) served as the reference group.

## Discussion

The primary finding of this large prospective investigation of over 225,000 women and men followed for nine years is that adiposity as assessed by WC was strongly and consistently related to death from a comprehensive list of death from major specific causes, including deaths from lung cancer and chronic respiratory disease, independent of BMI and other covariates. In contrast, adiposity as assessed by BMI showed inconsistent associations with specific causes of death, displaying positive relations to death from non-lung cancers, cardiovascular disease, and non-cancer/non-cardiovascular diseases, but inverse or null associations with deaths from lung cancer and chronic respiratory disease. This finding indicates that an increased amount of abdominal fat, but not general adiposity, represents a consistent predictor of premature death from major specific causes.

Our results have significant clinical implications because they suggest that WC represents a superior predictor than BMI of risk of deaths from lung cancer and chronic respiratory disease, two major causes of death. For example, obtaining a WC measurement in individuals already at increased risk for respiratory death (e.g., due to smoking or chronic obstructive respiratory disease) provides important information not delivered by BMI on a patient's risk of premature mortality.

Though speculative, one possible biologic reason for the heterogeneous associations of WC and BMI to mortality from lung cancer and chronic respiratory disease is that lung cancer mortality and chronic respiratory disease mortality may involve biologic pathways related to insulin resistance and related metabolic abnormalities, such as excess release of proinflammatory and prothrombotic factors [18], [19], which are more strongly related to abdominal adiposity than to overall excess body weight.

An alternative explanation for the divergent relations of WC and BMI to deaths from lung cancer and chronic respiratory disease is heterogeneity with respect to residual confounding by smoking. Current smoking is related to both lower BMI [20] and increased mortality from lung cancer and chronic respiratory disease. By comparison, current smoking is associated with visceral fat accumulation [21] and smoking cessation is related to increases in WC [22]. Limiting the investigation to participants who never smoked helps resolve the issue of residual confounding by smoking with respect to both BMI and WC but our cohort lacked sufficient numbers of cases of mortality from lung cancer and chronic respiratory disease among persons who never smoked.

To the best of our knowledge, no previous study has examined the association between WC and risk of death from lung cancer. However, our results regarding WC are consistent with previous epidemiologic investigations that have focused on lung cancer incidence. For example, the Iowa Women's Health Study found a positive relation of WC (relative risk comparing extreme categories = 1.76; 95 percent confidence interval, 1.14 to 2.73) to lung cancer incidence [23]. Similarly, the Women's Health Initiative observed positive relations of WC to lung cancer incidence among current smokers (relative risk = 1.56; 95 percent confidence interval, 0.91 to 2.69) and former smokers (relative risk = 1.50; 95 percent confidence interval, 0.98 to 2.31) [24].

Our findings regarding the relation of BMI to lung cancer death are strikingly consistent with recent data from a large pooled analysis of 57 prospective studies [25]. In that analysis, each 5kg/m^2^ increment in BMI was associated with a relative risk of lung cancer death of 0.71 (95 percent confidence interval, 0.63 to 0.79) within the BMI range of 15 to 25 kg/m^2^ and was associated with a relative risk of lung cancer death of 0.98 (95 percent confidence interval, 0.88 to 1.09) within the BMI range of 25 to 50 kg/m^2^ [25].

Our results of a monotonically positive relation of WC to respiratory death are comparable to recent data from the European Prospective Investigation into Cancer and Nutrition (EPIC) Study [26]. In that study, the relative risk of respiratory death comparing extreme quintiles of WC among women was 2.95 (95 percent confidence interval, 1.52 to 5.70) and the corresponding relative risk among men was 6.56 (95 percent confidence interval, 3.60 to 11.96).

Our results of a J-shaped association between BMI and death from chronic respiratory disease are comparable to the aforementioned pooled analysis of 57 prospective studies [25]. Each 5kg/m^2^ increase in BMI was related to a relative risk of respiratory death of 0.31 (95 percent confidence interval, 0.28 to 0.35) within the BMI range of 15 to 25 kg/m^2^ and was related to a relative risk of respiratory death of 1.20 (95 percent confidence interval, 1.07 to 1.34) within the BMI range of 25 to 50 kg/m^2^ [25].

Notable strengths of our study include its prospective design, a large number of deaths from specific causes, a high follow-up rate, and detailed information on potentially confounding factors. Despite a number of advantageous aspects of our study, one potential limitation is a low response rate to the second questionnaire used to obtain WC information, which could have resulted in selection bias if, for example WC was preferentially missing for persons with high mortality risk. A further potential limitation is that WC, weight, and height were assessed using self-report, a method that is known to be imperfect. However, self-reported BMI values and WC measures have been found to be sufficiently precise for use in epidemiologic studies [15], [16]. Self-reported height and weight vary systematically with BMI [27], but the importance of such bias is uncertain [28]. Taken together, it is improbable that misclassification of self-reported WC, height, and weight fully accounted for our finding of markedly divergent relations of general and abdominal adiposity to risk for deaths from lung cancer and chronic respiratory disease. We did not evaluate the association between WC and mortality from chronic lung disease according to smoking status because that topic is beyond the scope of the present investigation. Future studies should examine that relation.

We conclude that increased abdominal fat, but not general adiposity is associated with elevated risk of a comprehensive list of death from major specific causes, including deaths from lung cancer and chronic respiratory disease. Based on our observational epidemiologic data, deaths from lung cancer and chronic respiratory disease may potentially be added to the list of causes of death directly related to adiposity.
